# Avoidable waste related to inadequate methods and incomplete reporting of interventions: a systematic review of randomized trials performed in Sub-Saharan Africa

**DOI:** 10.1186/s13063-017-2034-0

**Published:** 2017-07-05

**Authors:** Lee Aymar Ndounga Diakou, Francine Ntoumi, Philippe Ravaud, Isabelle Boutron

**Affiliations:** 1 Fondation Congolaise pour la Recherche Médicale (FCRM), Brazzaville, Congo; 2 INSERM, UMR 1153 Epidemiology and Biostatistics Sorbonne Paris Cité Center (CRESS), METHODS Team, Paris, France; 3 Paris Descartes University, Paris, France; 4 Marien Ngouabi University, Brazzaville, Democratic Republic of the Congo; 5 Institute for Tropical Medicine, University of Tubingen, Tubingen, Germany; 6 Centre d’Épidémiologie Clinique, Hôpital Hôtel Dieu, Assistance Publique des Hôpitaux de Paris, Paris, France

**Keywords:** Sub-Saharan Africa, Randomized controlled trials, Risk of bias, Reporting, Research implementation

## Abstract

**Background:**

Randomized controlled trials (RCTs) are needed to improve health care in Sub-Saharan Africa (SSA). However, inadequate methods and incomplete reporting of interventions can prevent the transposition of research in practice which leads waste of research. The aim of this systematic review was to assess the avoidable waste in research related to inadequate methods and incomplete reporting of interventions in RCTs performed in SSA.

**Methods:**

We performed a methodological systematic review of RCTs performed in SSA and published between 1 January 2014 and 31 March 2015. We searched PubMed, the Cochrane library and the African Index Medicus to identify reports. We assessed the risk of bias using the Cochrane Risk of Bias tool, and for each risk of bias item, determined whether easy adjustments with no or minor cost could change the domain to low risk of bias. The reporting of interventions was assessed by using standardized checklists based on the Consolidated Standards for Reporting Trials, and core items of the Template for Intervention Description and Replication. Corresponding authors of reports with incomplete reporting of interventions were contacted to obtain additional information. Data were descriptively analyzed.

**Results:**

Among 121 RCTs selected, 74 (61%) evaluated pharmacological treatments (PTs), including drugs and nutritional supplements; and 47 (39%) nonpharmacological treatments (NPTs) (40 participative interventions, 1 surgical procedure, 3 medical devices and 3 therapeutic strategies). Overall, the randomization sequence was adequately generated in 76 reports (62%) and the intervention allocation concealed in 48 (39%). The primary outcome was described as blinded in 46 reports (38%), and incomplete outcome data were adequately addressed in 78 (64%). Applying easy methodological adjustments with no or minor additional cost to trials with at least one domain at high risk of bias could have reduced the number of domains at high risk for 24 RCTs (19%). Interventions were completely reported for 73/121 (60%) RCTs: 51/74 (68%) of PTs and 22/47 (46%) of NPTs. Additional information was obtained from corresponding authors for 11/48 reports (22%).

**Conclusion:**

Inadequate methods and incomplete reporting of published SSA RCTs could be improved by easy and inexpensive methodological adjustments and adherence to reporting guidelines.

**Electronic supplementary material:**

The online version of this article (doi:10.1186/s13063-017-2034-0) contains supplementary material, which is available to authorized users.

## Background

Sub-Saharan Africa (SSA) is characterized by extensive morbidity and mortality mainly due to infectious diseases as well as chronic and noncommunicable diseases [1, 2]. As the standard for establishing the effectiveness of two interventions or more, randomized controlled trials (RCTs) are needed to overcome the global burden of disease and to improve health care in SSA.

Results of RCTs performed in high-income countries (HICs) cannot easily be transposed to SSA because of differences of contexts [3, 4]. In fact, the social and cultural environment, infrastructure organization and availability of facilities differ greatly [5]. For instance, in SSA, people often consult a physician late, frequently use self-medication and have several comorbidities (malnourishment, anemia, malaria, etc.) and do not always adhere to treatment [6, 7].

Recently, Chalmers and colleagues raised an important debate by highlighting that up to 85% of current research is wasted and that most of this waste is avoidable [8]. For example, a recent study showed that 43% of RCTs included in Cochrane systematic reviews had at least one domain at high risk of bias; while simple methodological adjustments with no or minor cost could have been applied to reduce this risk [9]. For example, the use of sequentially numbered, opaque, sealed envelopes to conceal the treatment allocation or performing an intention-to-treat analysis are simple low-cost methods that would avoid bias [9]. Similarly, Glasziou and colleagues demonstrated that inadequate reporting of interventions for RCTs was frequently a barrier to the transposition of research results to practice [10, 11].

This avoidable waste in research raises an important concern because such RCTs are not consistent with the Declaration of Helsinki which requires that “Medical research involving human subjects must conform to generally accepted scientific principles, be based on a thorough knowledge of the scientific literature (…)” and that “Researchers have a duty to make publicly available the results of their research on human subjects and are accountable for the completeness and accuracy of their reports.”

In a first study on the epidemiology of published RCTs performed in SSA, we showed that most RCTs focused on diseases with a high burden in SSA. However, the leadership and funding sources of these trials were mainly from high-income countries (HICs) [12]. The objective of the current study was to assess the avoidable waste in research related to inadequate methods (i.e., those leading to a high risk of bias) and incomplete reporting of interventions for RCTs performed in SSA.

## Methods

This methodological systematic review was conducted and reported according to the Preferred Reporting Items for Systematic reviews and Meta-Analyses Statements (PRISMA) [13]. The checklist items pertain to the content of the review is available in Additional file 1. As the study did not concern human or clinical data, we did not record the protocol on PROSPERO.

### Searches

We performed a methodological systematic review of all RCTs conducted in SSA and published from 1 January 2014 to 31 March 2015. We relied on a sample of RCTs that was used in a previous work which aimed to describe the epidemiology of RCTs in SSA [13]. In brief, we searched for all reports of published RCTs indexed in PubMed, the Cochrane Central Register of Controlled Trials (CENTRAL) and the African Index Medicus (AIM) based on the Cochrane Highly Sensitive Search Strategy for identifying randomized trials [14] combined to a geographic search filter to identify RCTs in Africa [15]. The core search string was varied depending on the database (see Additional file 2). The AIM database was developed by the World Health Organization (WHO) in collaboration with the Association for Health Information and Libraries in Africa (AHILA). It gives access to information published in, or related to Africa and includes about 140 African journals [16]. The search strategy for the AIM database was carried out with the help of the head librarian of the WHO regional office for Africa (WHO-Afro). LAND searched PubMed and CENTRAL.

### Study inclusion and exclusion criteria

We downloaded all retrieved references in Endnote and all duplicates were deleted. First, one of us (LAND) screened all titles and abstracts to identify the relevant studies based on defined eligibility criteria. Another researcher (CL or RH or AB) confirmed the trials’ eligibility. If the information was unclear or insufficient in the abstract, the full-text article was systematically retrieved to confirm eligibility. Then, the same researcher (LAND) retrieved all full texts and assessed their eligibility. We included RCTs with at least one center located in SSA. International multicenter trials including both participants from SSA and non-SSA countries were also eligible. We defined an RCT as a clinical study that randomly allocated participants to different interventions: pharmacological treatments (PTs) including drugs or nutritional supplements; and nonpharmacological treatments (NPTs) such as education and training (e.g., exercise program), service delivery, rehabilitation, devices or surgery. We excluded reports of secondary publications of RCTs, phase I/II trials, pilot studies, nonrandomized and pseudo-randomized studies, observational studies, and reports of studies pooling data from more than one RCT. Protocols of RCTs, meeting abstracts, letters, comments and books were also excluded. We did not apply any language restriction for study eligibility.

### Data extraction strategy

Two review authors independently recorded data by using a standardized data extraction form. Disagreements were resolved by discussion and consensus. We systematically assessed and recorded the following:

1. General characteristics of RCT reports
  We recorded the study location, the medical area, study design, type of experimental interventions (PTs or NPTs) and the comparator, and sample size.
2. Risk of bias of the RCTs
  We used the Risk of Bias (RoB) tool developed by the Cochrane Collaboration [17] to assess the following key domains: sequence generation, allocation concealment, blinding of participants, care providers and outcome assessors, and incomplete outcome data. Two researchers (LAND and (CL or RH or AB)) independently assessed the risk of bias and then discussed the assessment to reach consensus. A third assessor (IB) was involved if needed. For each domain, we evaluated whether the risk of bias was high (i.e., may alter the results seriously), low (i.e., if present, unlikely to alter the results seriously), or unclear (i.e., insufficient information reported to permit judgement) [17]. The definition of the domains of the RoB tool and the support for judgement are reported in Table 1 [18].

**Table 1:** Definition of the domains of the Risk of Bias tool and the support for judgement according to the *Cochrane handbook for systematic reviews of interventions*

Domains (type of bias)	Review authors’ judgement		
	Low risk of bias	High risk of bias	Unclear risk of bias.
Random sequence generation (selection bias)	The investigators describe a random component in the sequence generation process such as drawing of lots	The investigators describe a nonrandom component in the sequence generation process. Usually, the description would involve some systematic, nonrandom approach	Insufficient information about the sequence generation process to permit judgement of “low risk” or “high risk”
Allocation concealment (selection bias)	Participants and investigators enrolling participants could not foresee assignment because of the use of, for example, sequentially numbered, opaque, sealed envelopes to conceal allocation	Participants or investigators enrolling participants could possibly foresee assignments and thus introduce selection bias, such as allocation based on assignment envelopes used without appropriate safeguards (e.g., if envelopes were unsealed or nonopaque or not sequentially numbered)	Insufficient information to permit judgement of “low risk” or “high risk”. This is usually the case if the method of concealment is not described or not described in sufficient detail to allow a definite judgement – for example, if the use of assignment envelopes is described, but it remains unclear whether envelopes were sequentially numbered, opaque and sealed
Blinding of participants and personnel (performance bias)	Any one of the following: • No blinding or incomplete blinding, but the review authors judge that the outcome is not likely to be influenced by lack of blinding • Blinding of participants and key study personnel ensured, and unlikely that the blinding could have been broken	Any one of the following: • No blinding or incomplete blinding, and the outcome is likely to be influenced by lack of blinding • Blinding of key study participants and personnel attempted but likely that the blinding could have been broken, and the outcome is likely to be influenced by lack of blinding	Any one of the following: • Insufficient information to permit judgement of “low risk” or “high risk” • The study did not address this outcome
Blinding of outcome assessment (detection bias)	Any one of the following: • No blinding of outcome assessment, but the review authors judge that the outcome measurement is not likely to be influenced by lack of blinding • Blinding of outcome assessment ensured, and unlikely that the blinding could have been broken	Any one of the following: • No blinding of outcome assessment, and the outcome measurement is likely to be influenced by lack of blinding • Blinding of outcome assessment, but likely that the blinding could have been broken, and the outcome measurement is likely to be influenced by lack of blinding	Any one of the following: • Insufficient information to permit judgement of “low risk” or “high risk” • The study did not address this outcome
Incomplete outcome data (attrition bias)	Any one of the following: • No missing outcome data • Reasons for missing outcome data unlikely to be related to true outcome (for survival data, censoring unlikely to be introducing bias) • Missing outcome data balanced in numbers across intervention groups, with similar reasons for missing data across groups • For dichotomous outcome data, the proportion of missing outcomes compared with observed event risk not enough to have a clinically relevant impact on the intervention effect estimate • For continuous outcome data, plausible effect size (difference in means or standardized difference in means) among missing outcomes not enough to have a clinically relevant impact on observed effect size • Missing data have been imputed using appropriate methods.	Any one of the following: • Reason for missing outcome data likely to be related to true outcome, with either imbalance in numbers or reasons for missing data across intervention groups • For dichotomous outcome data, the proportion of missing outcomes compared with observed event risk enough to induce clinically relevant bias in intervention effect estimate • For continuous outcome data, plausible effect size (difference in means or standardized difference in means) among missing outcomes enough to induce clinically relevant bias in observed effect size • “As-treated” analysis done with substantial departure of the intervention received from that assigned at randomization • Potentially inappropriate application of simple imputation.	Any one of the following: • Insufficient reporting of attrition/exclusions to permit judgement of “low risk” or “high risk” (e.g., number randomized not stated, no reasons for missing data provided) • The study did not address this outcome.
  For each trial with at least one domain at high risk of bias, we identified the methodological problem(s) and determined whether easy adjustments with no or minor cost could change the domains to low risk of bias according to the classification proposed by Yordanov and colleagues [9]. We focused on easy methodological adjustments with no or minor costs because of funding constraints related to research in the context of SSA. Minor cost was defined as ≤5% of the total cost of the trial [9]
3. Completeness of reporting characteristics of health care interventions
  To evaluate the completeness of reporting of the interventions, we used a standardized data extraction form based on the Consolidated Standards for Reporting Trials (CONSORT) statement, its extension for NPTs as well as core items of the Template for Intervention Description and Replication (TIDieR) Checklist [19–21]. The data extraction for each type of interventions is reported in Table 2.

**Table 2:** Extraction fields across different types of health care interventions

Extraction fields	Type of interventions			
	Pharmacological (drugs and nutritional supplements)	Nonpharmacological (rehabilitation, behavioral treatment, education, psychotherapy)	Surgical procedures and medical devices (disposal or implementable)	Other (e.g., therapeutic strategies)
Setting (location and type of infrastructure delivering the intervention)	x	x	x	x
Dose	x			
Mode of administration (e.g., oral versus intravenous)	x			
Timing	x			
Duration of treatment	x			
Treatment adherence	x	x		
Intervention development process		x		
Intervention content (components)		x		
Equipment or materials used or provided (physical or informational)		x		x
Mode of implementation (e.g., individually versus in groups)		x		
Schedule (frequency or intensity, timing and duration)		x		
Care provider background		x	x	x
Pre-(operative) care			x	x
Anesthesia			x	
Procedure (sequencing of the technique)			x	x
Post-(operative) care			x	x
  We considered an intervention completely reported when the fields listed in Table 2 were reported and when applicable [22]. Then, we systematically emailed the corresponding authors of reports with incomplete reporting of the intervention and asked them to send us any missing details. One reminder was systematically sent after 1 month of no reply. We concluded no response when any reply was received to that reminder 3 months later.
4. Endorsement of CONSORT by journals
  We systematically searched all journal’s websites for instructions to authors to check whether it required adherence to the CONSORT statement or to the EQUATOR Website.

### Data analysis

We entered the details of initial and follow-up ratings of reports into a customized Excel database. All analyses involved the use of SAS for Windows 9.3 (SAS Inst., Cary, NC, USA). For each item assessed, and by intervention type, data were summarized descriptively as frequency and percentage or median and interquartile range (IQR). We performed a post hoc analysis, using a chi-square test for categorical data, to assess the link between the endorsement of CONSORT by journals and the completeness of intervention reporting (alpha = 0.05).

## Results

### Reports identification

**Fig. 1:**
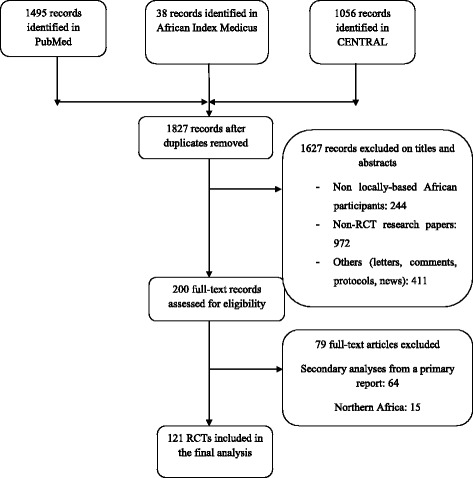
Flow diagram

The screening process is described in Fig. 1. The search identified 1827 reports after removing duplicates. The initial screening excluded 1627 records. For the remaining 200 reports, the full-text articles were retrieved for analysis. We declared 79 reports ineligible because they concerned secondary analyses of primary RCT reports (81%, *n* = 64) or were conducted in northern Africa (19%, *n* = 15). Overall, 121 reports were included for data extraction. All the selected references are reported in the Additional file 3.

### Study characteristics

General characteristics of the studies are in Table 3. Most RCTs focused on malaria (20%, *n* = 25), HIV/AIDS (19%, *n* = 24), tuberculosis (3.3%; *n* = 4), diarrheal diseases (2.4%; *n* = 3), preterm birth complications (1.6%; *n* = 2) and other diseases (52.2%, *n* = 63).

**Table 3:** General characteristics of the included randomized controlled trial reports

Characteristics	*N* = 121
Trial location	
Sub-Saharan countries only	104 (85.9)
• South Africa	20 (19.2)
• Nigeria	12 (11.5)
• Tanzania	10 (9.6)
• Kenya	8 (7.6)
• Uganda	8 (7.6)
• Malawi	6 (5.7)
• Rwanda	4 (3.8)
• Ethiopia	3 (2.8)
Several Sub-Saharan countries	5 (4.1)
Sub-Saharan African countries and high-income countries (HICs) or other countries (not HICs)	12 (8.9)
Medical area	
Malaria	25 (20.6)
HIV/AIDS	24 (19.9)
Tuberculosis	4 (3.3)
Diarrheal diseases	3 (2.4)
Preterm birth complications	2 (1.6)
Other diseases	63 (52.2)
Study design	
Parallel groups	97 (80.1)
Clusters	19 (15.8)
Factorial design	3 (2.4)
Cross-over	2 (1.7)
Experimental intervention	
Pharmacological (drugs and nutritional supplements)	74 (61.1)
Nonpharmacological	47 (38.9)
• Participative interventions	40 (85.1)
• Devices	3 (6.3)
• Surgical procedures	1 (2.3)
• Therapeutic strategies	3 (6.3)
Comparator	
Active treatment	43 (35.5)
Usual care	32 (26.4)
Placebo	24 (19.9)
Other	22 (18.2)
Sample size (median [IQR])	346 [160–932]

Data are number. (%) unless indicated. *IQR* interquartile range

The median (IQR) sample size was 346 [160–932]. The study design was mainly parallel individual groups (80%, *n* = 97) and cluster RCTs (15%, *n* = 19). Interventions evaluated both PTs (61%, *n* = 74) and NPTs (39%, *n* = 47), including participative interventions (85%, *n* = 40), surgical procedures (2%, *n* = 1), implementable or disposal devices (2%, *n* = 3) and therapeutic strategies (2%, *n* = 3). The comparator interventions were active interventions (35%, *n* = 43), usual care (26%, *n* = 32) or placebo (19%, *n* = 24).

### Inadequate methods and risk of bias in RCTs

**Fig. 2:**
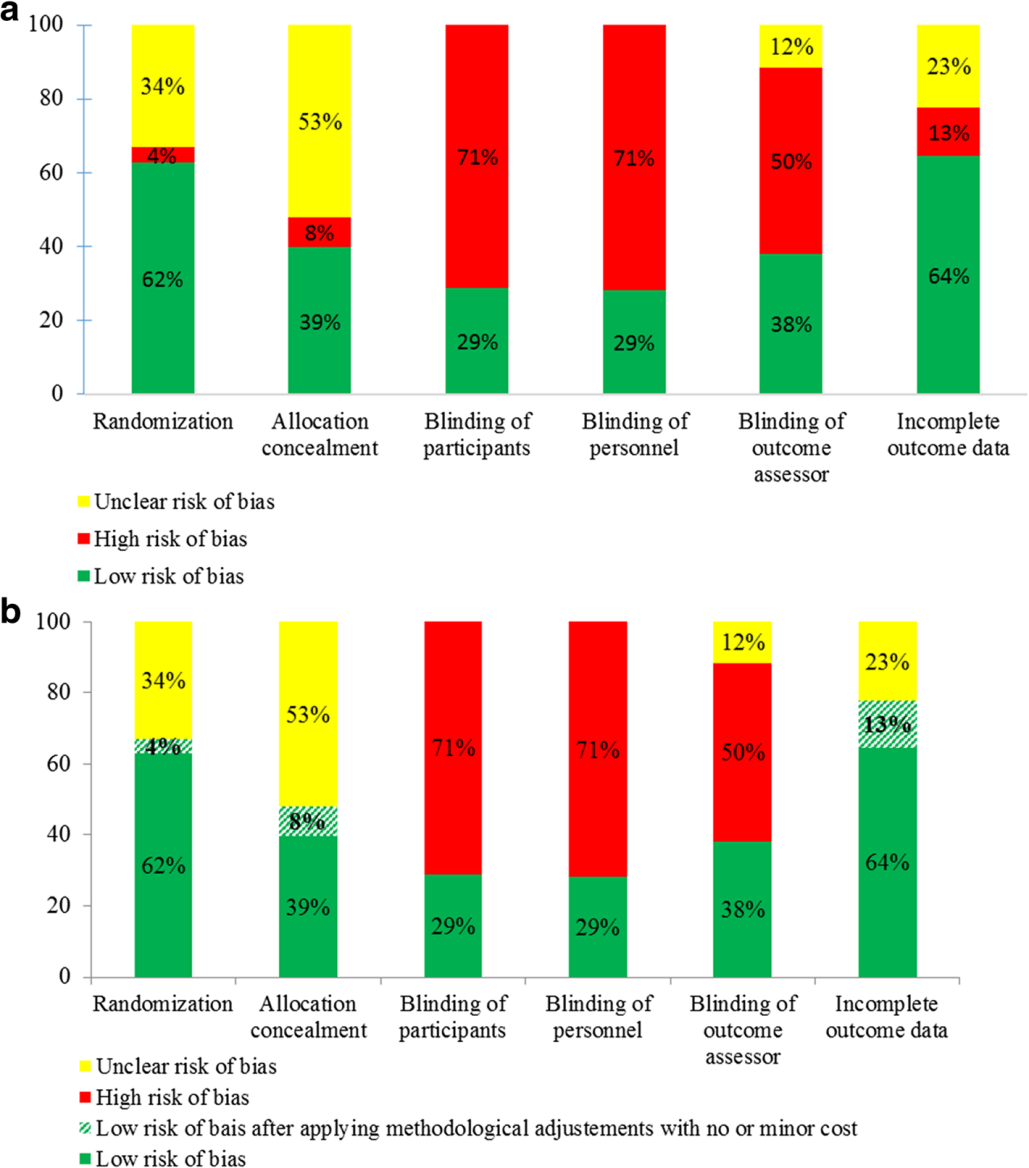
Risk of bias in 121 randomized controlled trials (RCTs) performed in Sub-Saharan Africa. Initial risk of bias (**a**) and risk of bias after applying easy methodological adjustments with no or minor additional cost (**b**). *Vertical bars* represent domains assessed according the Risk of Bias tool of the Cochrane Collaboration

The risk of bias in reports of RCTs is in Fig. 2. Overall, 92/121 trials (76%) had at least one domain at high risk of bias. Included RCTs were at high risk of bias for 5 (4%) for generation of randomization sequence, 10 (8%) for allocation concealment, 87 (71%) for both blinding of participants and personnel, and 61 (50%) for blinding of outcome assessor. Incomplete outcome data were inadequately addressed in 16 (13%).

Applying easy methodological adjustments with no or minor additional cost to trials with at least one domain at high risk of bias could have reduced the number of domains at high risk for 24 trials (19%). These adjustments could correct all trials at high risk of bias for sequence generation, allocation concealment and incomplete outcome data especially. None of the RCTs at high risk of bias for blinding status of participants, personnel or outcome assessors could be corrected because this would involve a medium or difficult adjustment with major cost [9]. All methodological adjustments applied to each identified problem according to the domain of risk of bias are described in Table 4.

**Table 4:** Problems identified in randomized controlled trials confirmed to be at high risk of bias and methodological adjustments with no or minor cost applied

Domains	Type of problem in original trial report	*N* = 121 No. (%)	Methodological adjustment	Cost
Generation of randomization sequence	Inappropriate randomization methods including sequence generated by some rule based on date/day of admission or on hospital or clinic record number	5 (4)	Referring to a random number table; Using a computer random-number generator Coin tossing Shuffling cards or envelopes Throwing dice Drawing of lots	No cost
Allocation concealment	No explicitly unconcealed procedure or unsealed or nonopaque or not sequentially numbered envelopes	10 (8)	Central allocation (including telephone, web-based and pharmacy-controlled randomization) or sequentially numbered, opaque, sealed envelopes	Minor
Incomplete outcome data	Exclusion of patients from the analysis	14 (11)	Intention-to-treat analysis	No cost
	Intention-to-treat analysis but inadequate missing data imputation	2 (1)	Intention-to-treat analysis with a multiple imputation method	Minor

### Completeness of the intervention reporting

**Fig. 3:**
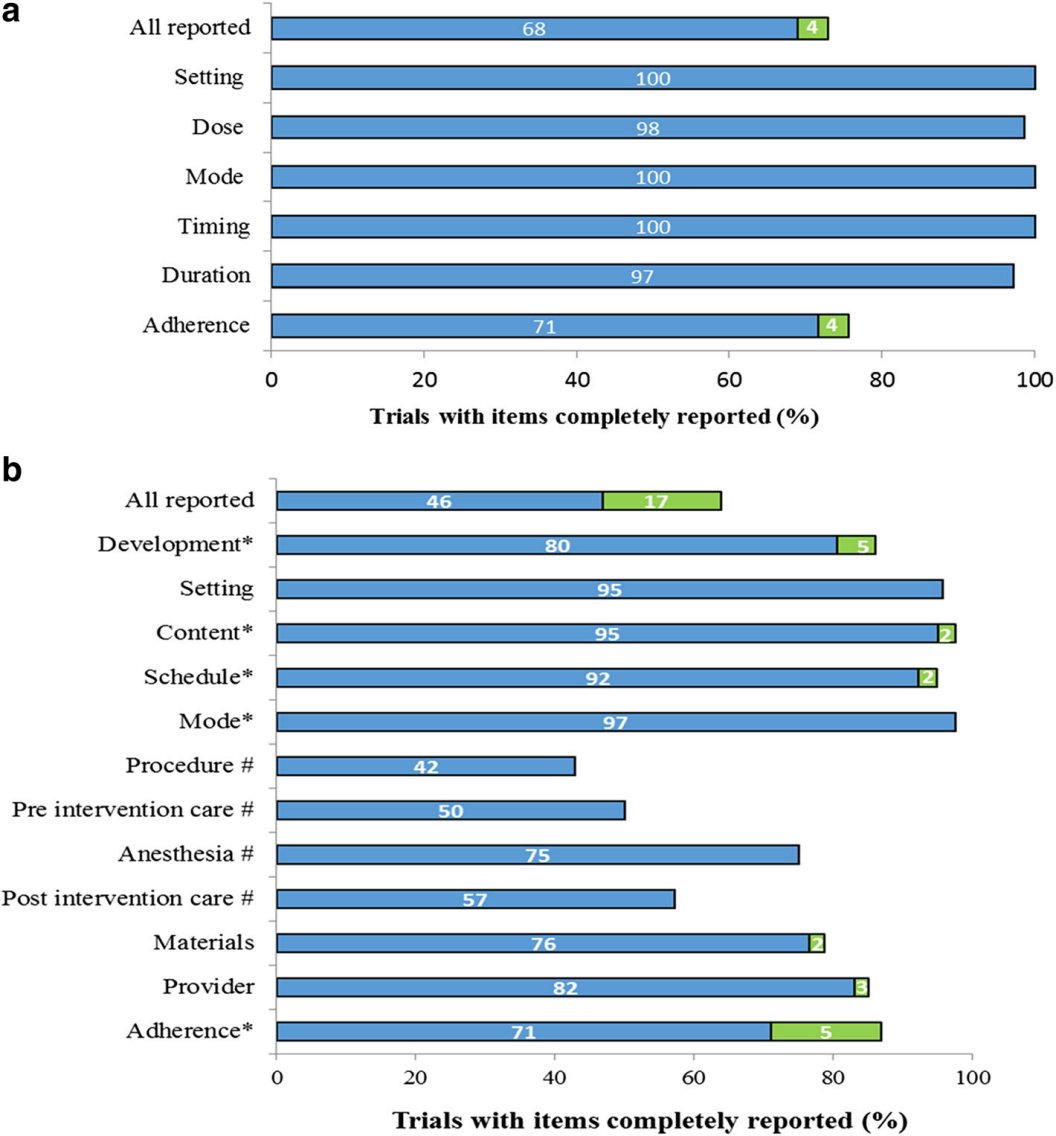
Reporting of interventions evaluated in randomized controlled trials (RCTs) performed in Sub-Saharan African (*n* = 121). Pharmacological treatments, *n* = 74 RCTs (**a**) and nonpharmacological treatments, *n* = 47 RCTs (**b**). For both type of interventions, the percentage of interventions rated as completely reported for each item in initial trial reports is illustrated in *blue*. The additional percentage after author reply is in *green*. (*) items assessed only for participative interventions. (#) items assessed only for devices or surgical procedures

Reporting of each intervention item for included RCTs is described in Fig. 3. The information needed was completely reported in 60% (*n* = 73/121) of the articles; 68% (*n* = 51/74) of PTs and 46% (*n* = 22/47) of NPTs. None of the RCTs evaluating surgical procedures (*n* = 1), medical devices (*n* = 3), or therapeutic strategies (*n* = 3) was completely reported. Request for additional information for reports with incomplete reporting provided data for 11/48 RCTs (22%).

Overall, we found that 68% (*n* = 82/121) of RCTs were published in a journal requiring adherence to CONSORT. Reporting was complete in 62% (*n* = 51/82) of RCTs published in journals requiring adherence to the guidelines versus 51% (*n* = 20/39) when published in a journal not requiring CONSORT adherence; chi^2^ = 1,29; *P* = 0.25.

## Discussion

We evaluated the avoidable waste in research related to inadequate methods and incomplete reporting of interventions in reports of RCTs performed in SSA and published over 1 year. Applying easy and methodological adjustments with no or minor cost could have limited the number of domains at high risk of bias for 19% of trials with at least one domain at high risk. Moreover, additional information requested of the corresponding authors of reports with incomplete descriptions overall improved the completeness of trial reporting from 60% to 69%.

### Comparison with other studies

Most studies evaluating the reporting and methods of RCTs conducted in SSA focused on specific diseases, and to our knowledge, none evaluated the impact of methodological adjustments [23–26]. In a review of 76 RCTs of HIV/AIDS conducted in Africa, the generation of the randomization sequence and the allocation concealment were judged adequate for only 41% and 51% of trials respectively, and 33% of trials reported blinded outcome assessors [24]. Another descriptive analysis of 60 African RCTs of malaria reported adequate generation of random sequence and allocation concealment for 58% and 23% respectively, with participants or providers adequately blinded in 38% and loss to follow-up accounted for in 81% of reports [26]. Even though results vary to some extent among studies, these reviews raised substantial issues related to inadequate methods in SSA trials.

Furthermore, the concern of poor trial reporting remains a long-told story [27, 28]. Although we did not find any study specifically focusing on how completely interventions are described in reports of SSA RCTs, the inadequate description of interventions in trial reports has been pointed out for many years [9, 11, 12]. In their early analysis, Glasziou and colleagues concluded that poor reporting was an important barrier to the replication of interventions in clinical practice [9]. Recently, Hoffmann and colleagues showed inadequate descriptions for more than 60% of NPTs [29]. In another cross-sectional study of published trials, Schröter and colleagues highlighted that the most poorly described aspects of interventions in trial reports were the actual procedures involved, including the sequencing of the technique (what happened, how and when) and the physical or informational materials used [30]. These results were confirmed in our analysis of trials conducted in SSA. To increase research value, we must develop specific tools and support accessibility to researchers based in SSA. Furthermore, we need to work on specific solutions and tools to decrease this waste in SSA.

### Perspectives and implications

The CONSORT statement was developed to improve the reporting of RCTs [19]. However, even if the quality of reporting for RCTs has significantly increased since the publication and the endorsement of CONSORT by many journals [31–33], the reporting of interventions remains insufficient, particularly for NPTs [34–36]. To address this issue, the extension of the CONSORT statement for NPTs and the TIDieR Checklist were developed to improve the implementation of interventions in clinical practice [20, 21]. Nevertheless, adherence to these reporting guidelines must be improved at different levels, first by helping authors adherence to the CONSORT statement when writing the first draft of the manuscript. Second, editors should require and enforce adherence to the CONSORT statement. Third, the completeness of reporting should be monitored at the peer-review process by the submission of checklists or other types of interventions such as the development of tools to combine the CONSORT checklist and its extensions [37].

The implications of this work are important for SSA because of the small number of RCTs performed in this part of the world [38], and the shortage of research resources. For this reason, waste must be addressed. In accordance previous works [9, 39], our results highlight that waste in RCTs in SSA could be avoided with simple and inexpensive methodological adjustments as well as a better reporting of interventions. Investigators should be informed of the feasibility of these adjustments and reporting guidelines when planning their trials and drafting their reports to limit the number of flaws in trial methods and poor descriptions of interventions at an early stage [10, 12, 40]. The Enhancing the QUAlity and Transparency Of health Research (EQUATOR) network is an international initiative created to improve the reliability and value of published health research literature by promoting transparent and accurate reporting and wider use of robust reporting guidelines (http://www.equator-network.org/). In our study, articles journals recommending reporting guidelines in their instructions to authors have a better description of interventions than those that did not recommend any reporting guidelines.

### Limitations

This study has limitations. First, our results are based on a sample of published RCTs over 1 year, which could limit generalization. Although our search strategy was large, we cannot ensure that we identified all published RCTs performed in SSA. Second, the assessment of methods and reporting quality relies on what was reported in the published RCT reports, and a gap could exist between what was reported and what was done.

## Conclusion

Inadequate methods and incomplete reporting of published RCTs performed in SSA could be improved by easy and inexpensive methodological adjustments and adherence to reporting guidelines.

## Additional files

Additional file 1: PRISMA Checklist. (DOC 64 kb)

Additional file 2: Search strategy for randomized controlled trials’ (RCTs) reports. (DOC 26 kb)

Additional file 3: References for the selected randomized controlled trials’ (RCTs) reports. (DOC 128 kb)

## Abbreviations

AHILA: Association for Health Information and Libraries in Africa
AIM: African Index Medicus
CONSORT: Consolidated Standards of Reporting Trials
EQUATOR: Enhancing the QUAlity and Transparency Of health Research
HICs: High-income countries
HIV/AIDS: Human immunodeficiency virus/acquired immunodeficiency syndrome
IQR: Interquartile range
NPTs: Nonpharmacological treatments
PTs: Pharmacological treatments
RCT: Randomized controlled trial
RoB: Risk of bias
SSA: Sub-Saharan Africa
TIDieR: Template for Intervention Description and Replication
WHO: World Health Organization
