# Correction to: Avoidable waste related to inadequate methods and incomplete reporting of interventions: a systematic review of randomized trials performed in Sub-Saharan Africa

**DOI:** 10.1186/s13063-017-2431-4

**Published:** 2018-01-16

**Authors:** Lee Aymar Ndounga Diakou, Francine Ntoumi, Philippe Ravaud, Isabelle Boutron

**Affiliations:** 1 Fondation Congolaise pour la Recherche Médicale (FCRM), Brazzaville, Congo; 2 INSERM, UMR 1153 Epidemiology and Biostatistics Sorbonne Paris Cité Center (CRESS), METHODS Team, Paris, France; 3 Paris Descartes University, Paris, France; 4 Marien Ngouabi University, Brazzaville, Democratic Republic of the Congo; 5 Institute for Tropical Medicine, University of Tubingen, Tubingen, Germany; 6 Centre d’Épidémiologie Clinique, Hôpital Hôtel Dieu, Assistance Publique des Hôpitaux de Paris, Paris, France

## Correction

In the original publication [1] the last sentence in the last paragraph under ‘Perspectives and implications’ in the Discussions section needs to be removed. The correct version can be found in this Erratum.

Incorrect version:

The implications of this work are important for SSA because of the small number of RCTs performed in this part of the world [38], and the shortage of research resources. For this reason, waste must be addressed. In accordance previous works [9, 39], our results highlight that waste in RCTs in SSA could be avoided with simple and inexpensive methodological adjustments as well as a better reporting of interventions. Investigators should be informed of the feasibility of these adjustments and reporting guidelines when planning their trials and drafting their reports to limit the number of flaws in trial methods and poor descriptions of interventions at an early stage [10, 12, 40]. The Enhancing the QUAlity and Transparency Of health Research (EQUATOR) network is an international initiative created to improve the reliability and value of published health research literature by promoting transparent and accurate reporting and wider use of robust reporting guidelines (http://www.equator-network.org/). In our study, articles journals recommending reporting guidelines in their instructions to authors have a better description of interventions than those that did not recommend any reporting guidelines.

Correct version:

The implications of this work are important for SSA because of the small number of RCTs performed in this part of the world [38], and the shortage of research resources. For this reason, waste must be addressed. In accordance previous works [9, 39], our results highlight that waste in RCTs in SSA could be avoided with simple and inexpensive methodological adjustments as well as a better reporting of interventions. Investigators should be informed of the feasibility of these adjustments and reporting guidelines when planning their trials and drafting their reports to limit the number of flaws in trial methods and poor descriptions of interventions at an early stage [10, 12, 40]. The Enhancing the QUAlity and Transparency Of health Research (EQUATOR) network is an international initiative created to improve the reliability and value of published health research literature by promoting transparent and accurate reporting and wider use of robust reporting guidelines (http://www.equator-network.org/).
