# Supplementary material for: Avoidable waste related to inadequate methods and incomplete reporting of interventions: a systematic review of randomized trials performed in Sub-Saharan Africa
Source: Trials. 2017 Jul 5;18:291. doi: 10.1186/s13063-017-2034-0 (PMC5497345; doi:10.1186/s13063-017-2034-0)
Supplement: Supplementary file 2 — Search strategy for randomized controlled trials’ (RCTs) reports. (DOC 26 kb) [file 13063_2017_2034_MOESM2_ESM.doc]

**Search strategy for randomized controlled trial (RCT) reports**

1. Cochrane Highly Sensitive Search Strategy for identifying randomized trials in MEDLINE: sensitivity-maximizing version (2008 revision); PubMed format

1# “randomized controlled trial” [pt]

2# “controlled clinical trial” [pt]

3# “randomized” [tiab]

4# “placebo” [tiab]

5# “drug therapy” [sh]

6# “randomly” [tiab]

7# “trial” [tiab]

8# “groups” [tiab]

9#1 OR 2 OR 3 OR 4 OR 5 OR 6 OR 7 OR 8

10# ("Animals" [Mesh]) NOT "humans" [Mesh]

11# 9 NOT 10

1. Africa search string

("Africa"[MeSH] OR Africa*[tw] OR Angola[tw] OR Benin[tw] OR Botswana[tw] OR "Burkina Faso"[tw] OR Burundi[tw] OR Cameroon[tw] OR "Cape Verde"[tw] OR "Central African Republic"[tw] OR Chad[tw] OR Comoros[tw] OR Congo[tw] OR "Democratic Republic of Congo"[tw] OR Djibouti[tw] OR "Equatorial Guinea"[tw] OR Eritrea[tw] OR Ethiopia[tw] OR Gabon[tw] OR Gambia[tw] OR Ghana[tw] OR Guinea[tw] OR "Guinea Bissau"[tw] OR "Ivory Coast"[tw] OR "Cote d’Ivoire"[tw] OR Kenya[tw] OR Lesotho[tw] OR Liberia[tw] OR Madagascar[tw] OR Malawi[tw] OR Mali[tw] OR Mauritania[tw] OR Mauritius[tw] OR Mayote[tw] OR Mozambique[tw] OR Mocambique[tw] OR Namibia[tw] OR Niger[tw] OR Nigeria[tw] OR Principe[tw] OR Reunion[tw] OR Rwanda[tw] OR "Sao Tome"[tw] OR Senegal[tw] OR Seychelles[tw] OR "Sierra Leone"[tw] OR Somalia[tw] OR "South Africa"[tw] OR "St Helena"[tw] OR Sudan[tw] OR Swaziland[tw] OR Tanzania[tw] OR Togo[tw] OR Uganda[tw] OR Zaire[tw] OR Zambia[tw] OR Zimbabwe[tw] OR "Central Africa"[tw] OR "Central African"[tw] OR "West Africa"[tw] OR "West African"[tw] OR "Western Africa"[tw] OR "Western African"[tw] OR "East Africa"[tw] OR "East African"[tw] OR "Eastern Africa"[tw] OR "Eastern African"[tw] OR "South African"[tw] OR "Southern Africa"[tw] OR "Southern African"[tw] OR "sub Saharan Africa"[tw] OR "sub Saharan African"[tw] OR "subSaharan Africa"[tw] OR "subSaharan African"[tw]) NOT ("guinea pig"[tw] OR "guinea pigs"[tw] OR "aspergillus niger"[tw])

1. Final search strategy adapted and implemented according database

(((((((((((“randomized controlled trial” [Publication Type]) OR “controlled clinical trial” [Publication Type]) OR” randomized” [Title/Abstract]) OR “placebo” [Title/Abstract]) OR” drug therapy” [MeSH Subheading]) OR “randomly” [Title/Abstract]) OR “trial” [Title/Abstract]) OR “groups” [Title/Abstract])) NOT ((animals [MeSH Terms]) NOT humans [MeSH Terms]))) AND ((Africa [MeSH Terms]) OR Sub-Saharan Africa [MeSH Terms])
