# Supplementary material for: Avoidable waste related to inadequate methods and incomplete reporting of interventions: a systematic review of randomized trials performed in Sub-Saharan Africa
Source: Trials. 2017 Jul 5;18:291. doi: 10.1186/s13063-017-2034-0 (PMC5497345; doi:10.1186/s13063-017-2034-0)
Supplement: Supplementary file 3 — References for the selected randomized controlled trials’ (RCTs) reports. (DOC 128 kb) [file 13063_2017_2034_MOESM3_ESM.doc]

**References for the selected randomized controlled trial (RCTs) reports**

**A. Pharmacological treatments**

1. Abdallah, T. M., K. A. Elmardi, et al. Comparison of artesunate and quinine in the treatment of severe Plasmodium falciparum malaria at Kassala hospital, Sudan. J Infect Dev Ctries 2014; 8(5): 611-615.
2. Adegnika, A. A., J. F. Zinsou, et al. Randomized, controlled, assessor-blind clinical trial to assess the efficacy of single-versus repeated-dose albendazole to treat ascaris lumbricoides, trichuris trichiura, and hookworm infection. [Antimicrob Agents Chemother.](http://www.ncbi.nlm.nih.gov/pubmed/?term=Randomized%2C+controlled%2C+assessor-blind+clinical+trial+to+assess+the+efficacy+of+single-versus+repeated-dose+albendazole+to+treat+ascaris+lumbricoides%2C+trichuris+trichiura%2C+and+hookworm+infection) 2014 May; 58(5):2535-40. doi: 10.1128/AAC.01317-13.
3. Adeniyi, A. A., A. A. Odukogbe, et al. Randomization of two dosing regimens of vaginal misoprostol for cervical ripening and labor induction in a low resource setting. [Niger J Clin Pract.](http://www.ncbi.nlm.nih.gov/pubmed/?term=Randomization+of+two+dosing+regimens+of+vaginal+misoprostol+for+cervical+ripening+and+labor+induction+in+a+low+resource+setting.) 2014 May-Jun; 17(3):287-91. doi: 10.4103/1119-3077.130227.
4. Adjei, G. O., B. Q. Goka, et al. A randomized trial of artesunate-amodiaquine versus artemether-lumefantrine in Ghanaian paediatric sickle cell and non-sickle cell disease patients with acute uncomplicated malaria. [Malar J.](http://www.ncbi.nlm.nih.gov/pubmed/?term=A+randomized+trial+of+artesunate-amodiaquine+versus+artemether-lumefantrine+in+Ghanaian+paediatric+sickle+cell+and+non-sickle+cell+disease+patients+with+acute+uncomplicated+malaria) 2014 Sep 19; 13:369. doi: 10.1186/1475-2875-13-369.
5. Aebi, S., S. Gelber, et al. Chemotherapy for isolated locoregional recurrence of breast cancer (CALOR): a randomised trial. [Lancet Oncol.](http://www.ncbi.nlm.nih.gov/pubmed/?term=Chemotherapy+for+isolated+locoregional+recurrence+of+breast+cancer+(CALOR)%3A+a+randomised+trial) 2014 Feb; 15(2):156-63. doi: 10.1016/S1470-2045(13)70589-8.
6. Ashley, E. A., M. Dhorda, et al. Spread of artemisinin resistance in Plasmodium falciparum malaria. [N Engl J Med.](http://www.ncbi.nlm.nih.gov/pubmed/25075834) 2014 Jul 31; 371(5):411-23. doi: 10.1056/NEJMoa1314981.
7. Ashorn, P., L. Alho, et al. The impact of lipid-based nutrient supplement provision to pregnant women on newborn size in rural Malawi: a randomized controlled trial. [Am J Clin Nutr.](http://www.ncbi.nlm.nih.gov/pubmed/?term=The+impact+of+lipid-based+nutrient+supplement+provision+to+pregnant+women+on+newborn+size+in+rural+Malawi%3A+a+randomized+controlled+trial) 2015 Feb; 101(2):387-97. doi: 10.3945/ajcn.114.088617.
8. Awadzi, K., N. O. Opoku, et al. A Randomized, Single-Ascending-Dose, Ivermectin-Controlled, Double-Blind Study of Moxidectin in Onchocerca volvulus Infection. [PLoS Negl Trop Dis.](http://www.ncbi.nlm.nih.gov/pubmed/?term=A+Randomized%2C+Single-Ascending-Dose%2C+Ivermectin-Controlled%2C+Double-Blind+Study+of+Moxidectin+in+Onchocerca+volvulus+Infection) 2014 Jun 26; 8(6): e2953. doi: 10.1371/journal.pntd.0002953.
9. Benn, C. S., B. R. Diness, et al. Two different doses of supplemental vitamin A did not affect mortality of normal-birth-weight neonates in Guinea-Bissau in a randomized controlled trial. [J Nutr.](http://www.ncbi.nlm.nih.gov/pubmed/?term=J+Nutr+144(9)%3A+1474-1479) 2014 Sep; 144(9):1474-9. doi: 10.3945/jn.114.192674.
10. Boulware, D. R., D. B. Meya, et al. Timing of antiretroviral therapy after diagnosis of cryptococcal meningitis. [N Engl J Med.](http://www.ncbi.nlm.nih.gov/pubmed/?term=N+Engl+J+Med+370(26)%3A+2487-2498.) 2014 Jun 26; 370(26):2487-98. doi: 10.1056/NEJMoa1312884.
11. Chagaluka, G., C. Stanley, et al. Kaposi's sarcoma in children: an open randomised trial of vincristine, oral etoposide and a combination of vincristine and bleomycin. [Eur J Cancer.](http://www.ncbi.nlm.nih.gov/pubmed/?term=Eur+J+Cancer+50(8)%3A+1472-1481.) 2014 May; 50(8):1472-81. doi: 10.1016/j.ejca.2014.02.019.
12. Danno, K., F. Rerolle, et al. China rubra for side-effects of quinine: A prospective, randomised study in pregnant women with malaria in Cotonou, Benin. [Homeopathy.](http://www.ncbi.nlm.nih.gov/pubmed/?term=China+rubra+for+side-effects+of+quinine%3A+A+prospective%2C+randomised+study+in+pregnant+women+with+malaria+in+Cotonou%2C+Benin) 2014 Jul; 103(3):165-71. doi: 10.1016/j.homp.2014.03.002.
13. Djalle, D., S. P. Njuimo, et al. Efficacy and safety of artemether + lumefantrine, artesunate + sulphamethoxypyrazine-pyrimethamine and artesunate + amodiaquine and sulphadoxine-pyrimethamine + amodiaquine in the treatment of uncomplicated falciparum malaria in Bangui, Central African Republic: a randomized trial. [Malar J.](http://www.ncbi.nlm.nih.gov/pubmed?term=Djalle%5BAuthor%5D AND "Malar J"%5BJournal%5D AND 2014%5BDate - Publication%5D) 2014 Jan 7; 13:9. doi: 10.1186/1475-2875-13-9.
14. Ezeama, C. O., G. U. Eleje, et al. A comparison of prophylactic intramuscular ergometrine and oxytocin for women in the third stage of labor. [Int J Gynaecol Obstet.](http://www.ncbi.nlm.nih.gov/pubmed/24365208) 2014 Jan; 124(1):67-71. doi: 10.1016/j.ijgo.2013.07.020.
15. Eziefula, A. C., T. Bousema, et al. Single dose primaquine for clearance of Plasmodium falciparum gametocytes in children with uncomplicated malaria in Uganda: a randomised, controlled, double-blind, dose-ranging trial. [Lancet Infect Dis.](http://www.ncbi.nlm.nih.gov/pubmed/?term=Lancet+Infect+Dis+14(2)%3A+130-139.) 2014 Feb; 14(2):130-9. doi: 10.1016/S1473-3099(13)70268-8.
16. Falade, C. O., H. O. Dada-Adegbola, et al. Evaluation of the comparative efficacy and safety of artemether-lumefantrine, artesunate-amodiaquine and artesunate-amodiaquine-chlorpheniramine (Artemoclo) for the treatment of acute uncomplicated malaria in Nigerian children. [Med Princ Pract.](http://www.ncbi.nlm.nih.gov/pubmed/?term=Med+Princ+Pract+23(3)%3A+204-211.) 2014; 23(3):204-11. doi: 10.1159/000360578.
17. Fataki, M. R., R. R. Kisenge, et al. Effect of zinc supplementation on duration of hospitalization in Tanzanian children presenting with acute pneumonia. [J Trop Pediatr.](http://www.ncbi.nlm.nih.gov/pubmed/?term=Effect+of+zinc+supplementation+on+duration+of+hospitalization+in+Tanzanian+children+presenting+with+acute+pneumonia) 2014 Apr; 60(2):104-11. doi: 10.1093/tropej/fmt089.
18. Feikin, D. R., G. Bigogo, et al. Village-randomized clinical trial of home distribution of zinc for treatment of childhood diarrhea in rural Western kenya. [PLoS One.](http://www.ncbi.nlm.nih.gov/pubmed/?term=PLoS+One+9(5)%3A+e94436.) 2014 May 16; 9(5): e94436. doi: 10.1371/journal.pone.0094436.
19. Fisker, A. B., C. Bale, et al. High-dose vitamin A with vaccination after 6 months of age: a randomized trial. [Pediatrics.](http://www.ncbi.nlm.nih.gov/pubmed/?term=Pediatrics+134(3)%3A+e739-748.) 2014 Sep; 134(3): e739-48. doi: 10.1542/peds.2014-0550.
20. Flax, V. L., M. E. Bentley, et al. Plasma and breast-milk selenium in HIV-infected Malawian mothers are positively associated with infant selenium status but are not associated with maternal supplementation: results of the Breastfeeding, Antiretrovirals, and Nutrition study. [Am J Clin Nutr.](http://www.ncbi.nlm.nih.gov/pubmed/?term=Am+J+Clin+Nutr+99(4)%3A+950-956.) 2014 Apr; 99(4):950-6. doi: 10.3945/ajcn.113.073833.
21. Fleischhacker, W. W., R. Sanchez, et al. Aripiprazole once-monthly for treatment of schizophrenia: Double-blind, randomised, non-inferiority study. [Br J Psychiatry.](http://www.ncbi.nlm.nih.gov/pubmed/?term=Aripiprazole+once-monthly+for+treatment+of+schizophrenia%3A+Double-blind%2C+randomised%2C+non-inferiority+study) 2014 Aug; 205(2):135-44. doi: 10.1192/bjp.bp.113.134213.
22. Fuchs, C. S., J. Tomasek, et al. Ramucirumab monotherapy for previously treated advanced gastric or gastro-oesophageal junction adenocarcinoma (REGARD): an international, randomised, multicentre, placebo-controlled, phase 3 trial. [Lancet.](http://www.ncbi.nlm.nih.gov/pubmed/?term=Lancet+383(9911)%3A+31-39.) 2014 Jan 4; 383(9911):31-9. doi: 10.1016/S0140-6736(13)61719-5.
23. Gonzalez, R., G. Mombo-Ngoma, et al. Intermittent Preventive Treatment of Malaria in Pregnancy with Mefloquine in HIV-Negative Women: A Multicentre Randomized Controlled Trial. [PLoS Med.](http://www.ncbi.nlm.nih.gov/pubmed/?term=Intermittent+Preventive+Treatment+of+Malaria+in+Pregnancy+with+Mefloquine+in+HIV-Negative+Women%3A+A+Multicentre+Randomized+Controlled+Trial) 2014 Sep 23; 11(9): e1001733. doi: 10.1371/journal.pmed.1001733.
24. Grossman, D., D. Constant, et al. A randomized trial of misoprostol versus laminaria before dilation and evacuation in South Africa. [Contraception.](http://www.ncbi.nlm.nih.gov/pubmed/?term=A+randomized+trial+of+misoprostol+versus+laminaria+before+dilation+and+evacuation+in+South+Africa) 2014 Sep; 90(3):234-41. doi: 10.1016/j.contraception.2014.05.003.
25. Halliday, K. E., G. Okello, et al. Impact of intermittent screening and treatment for malaria among school children in Kenya: a cluster randomised trial. [PLoS Med.](http://www.ncbi.nlm.nih.gov/pubmed/?term=PLoS+Med+11(1)%3A+e1001594.) 2014 Jan 28; 11(1): e1001594. doi: 10.1371/journal.pmed.1001594.
26. Hammitt, L. L., J. Ojal, et al. Immunogenicity, impact on carriage and reactogenicity of 10-valent pneumococcal non-typeable Haemophilus influenzae protein D conjugate vaccine in Kenyan children aged 1-4 years: a randomized controlled trial. [PLoS One.](http://www.ncbi.nlm.nih.gov/pubmed/?term=PLoS+One+9(1)%3A+e85459.) 2014 Jan 21; 9(1): e85459. doi: 10.1371/journal.pone.0085459.
27. Idehen, H. O., F. E. Amadasun, et al. Comparison of intravenous colloid and colloid-crystalloid combination in hypotension prophylaxis during spinal anesthesia for cesarean section. [Niger J Clin Pract.](http://www.ncbi.nlm.nih.gov/pubmed/?term=Comparison+of+intravenous+colloid+and+colloid-crystalloid+combination+in+hypotension+prophylaxis+during+spinal+anesthesia+for+cesarean+section.) 2014 May-Jun; 17(3):309-13. doi: 10.4103/1119-3077.130231.
28. Johnson, J. L., H. Geldenhuys, et al. Effect of isoniazid therapy for latent TB infection on QuantiFERON-TB gold in-tube responses in adults with positive tuberculin skin test results in a high TB incidence area: a controlled study. [Chest.](http://www.ncbi.nlm.nih.gov/pubmed/?term=Chest+145(3)%3A+612-617) 2014 Mar 1; 145(3):612-7. doi: 10.1378/chest.13-1232.
29. Jindani, A., T. S. Harrison, et al. High-dose rifapentine with moxifloxacin for pulmonary tuberculosis. [N Engl J Med.](http://www.ncbi.nlm.nih.gov/pubmed/?term=N+Engl+J+Med+371(17)%3A+1599-1608.) 2014 Oct 23; 371(17):1599-608. doi: 10.1056/NEJMoa1314210.
30. Joray, M. L., T. W. Yu, et al. Zinc supplementation reduced DNA breaks in Ethiopian women. [Nutr Res.](http://www.ncbi.nlm.nih.gov/pubmed/?term=Zinc+supplementation+reduced+DNA+breaks+in+Ethiopian+women) 2015 Jan; 35(1):49-55. doi: 10.1016/j.nutres.2014.10.006.
31. Juan-Giner, A., C. Domicent, et al. A cluster randomized non-inferiority field trial on the immunogenicity and safety of tetanus toxoid vaccine kept in controlled temperature chain compared to cold chain. [Vaccine.](http://www.ncbi.nlm.nih.gov/pubmed/?term=A+cluster+randomized+non-inferiority+field+trial+on+the+immunogenicity+and+safety+of+tetanus+toxoid+vaccine+kept+in+controlled+temperature+chain+compared+to+cold+chain) 2014 Oct 29; 32(47):6220-6. doi: 10.1016/j.vaccine.2014.09.027.
32. Malu, C. K. K., D. M. Kahamba, et al. Efficacy of sublingual lorazepam versus intrarectal diazepam for prolonged convulsions in sub-saharan africa. [J Child Neurol.](http://www.ncbi.nlm.nih.gov/pubmed/?term=Efficacy+of+sublingual+lorazepam+versus+intrarectal+diazepam+for+prolonged+convulsions+in+sub-saharan+africa) 2014 Jul; 29(7):895-902. doi: 10.1177/0883073813493501.
33. Keiser, J., K. D. Silue, et al. Praziquantel, Mefloquine-Praziquantel, and Mefloquine-Artesunate-Praziquantel against Schistosoma haematobium: A Randomized, Exploratory, Open-Label Trial. [PLoS Negl Trop Dis.](http://www.ncbi.nlm.nih.gov/pubmed/?term=Praziquantel%2C+Mefloquine-Praziquantel%2C+and+Mefloquine-Artesunate-Praziquantel+against+Schistosoma+haematobium%3A+A+Randomized%2C+Exploratory%2C+Open-Label+Trial) 2014 Jul 17; 8(7): e2975. doi: 10.1371/journal.pntd.0002975.
34. Khalil, E. A., T. Weldegebreal, et al. Safety and efficacy of single dose versus multiple doses of AmBisome for treatment of visceral leishmaniasis in eastern Africa: a randomised trial. [PLoS Negl Trop Dis.](http://www.ncbi.nlm.nih.gov/pubmed/?term=PLoS+Negl+Trop+Dis+8(1)%3A+e2613.) 2014 Jan 16; 8(1): e2613. doi: 10.1371/journal.pntd.0002613.
35. Kiondo, P., G. Wamuyu-Maina, et al. The effects of vitamin C supplementation on pre-eclampsia in Mulago Hospital, Kampala, Uganda: A randomized placebo controlled clinical trial. [BMC Pregnancy Childbirth.](http://www.ncbi.nlm.nih.gov/pubmed/?term=The+effects+of+vitamin+C+supplementation+on+pre-eclampsia+in+Mulago+Hospital%2C+Kampala%2C+Uganda%3A+A+randomized+placebo+controlled+clinical+trial) 2014 Aug 21; 14:283. doi: 10.1186/1471-2393-14-283.
36. Klement, E., P. Pitche, et al. Effectiveness of co-trimoxazole to prevent plasmodium falciparum malaria in HIV-positive pregnant women in sub-saharan Africa: An open-label randomized controlled trial. [Clin Infect Dis.](http://www.ncbi.nlm.nih.gov/pubmed/24336820) 2014 Mar; 58(5):651-9. doi: 10.1093/cid/cit806.
37. Koegelenberg, C. F., F. Noor, et al. Efficacy of varenicline combined with nicotine replacement therapy vs varenicline alone for smoking cessation: a randomized clinical trial. [JAMA.](http://www.ncbi.nlm.nih.gov/pubmed/?term=JAMA+312(2)%3A+155-161.) 2014 Jul; 312(2):155-61. doi: 10.1001/jama.2014.7195.
38. Kosiborod, M., H. S. Rasmussen, et al. Effect of sodium zirconium cyclosilicate on potassium lowering for 28 days among outpatients with hyperkalemia: the HARMONIZE randomized clinical trial. [JAMA.](http://www.ncbi.nlm.nih.gov/pubmed/?term=JAMA+312(21)%3A+2223-2233.) 2014 Dec 3; 312(21):2223-33. doi: 10.1001/jama.2014.15688.
39. Kutamba, E., E. Mupere, et al. Dextrose boluses versus burette dextrose infusions in prevention of hypoglycemia among preterms admitted at Mulago Hospital: An open label randomized clinical trial. [Afr Health Sci.](http://www.ncbi.nlm.nih.gov/pubmed/?term=Dextrose+boluses+versus+burette+dextrose+infusions+in+prevention+of+hypoglycemia+among+preterms+admitted+at+Mulago+Hospital%3A+An+open+label+randomized+clinical+trial) 2014 Sep; 14(3):502-9. doi: 10.4314/ahs. v14i3.2.
40. Madhi, S. A., C. L. Cutland, et al. Influenza vaccination of pregnant women and protection of their infants. [N Engl J Med.](http://www.ncbi.nlm.nih.gov/pubmed/?term=N+Engl+J+Med+371(10)%3A+918-931.) 2014 Sep 4; 371(10):918-31. doi: 10.1056/NEJMoa1401480.
41. Manyando, C., E. M. Njunju, et al. Safety of daily co-trimoxazole in pregnancy in an area of changing malaria epidemiology: a phase 3b randomized controlled clinical trial. [PLoS One.](http://www.ncbi.nlm.nih.gov/pubmed/?term=PLoS+One+9(5)%3A+e96017.) 2014 May 15; 9(5): e96017. doi: 10.1371/journal.pone.0096017.
42. Marrazzo, J. M., G. Ramjee, et al. Tenofovir-based preexposure prophylaxis for HIV infection among African women. [N Engl J Med.](http://www.ncbi.nlm.nih.gov/pubmed/?term=N+Engl+J+Med+372(6)%3A+509-518.) 2015 Feb 5; 372(6):509-18. doi: 10.1056/NEJMoa1402269.
43. Merle, C. S., K. Fielding, et al. A four-month gatifloxacin-containing regimen for treating tuberculosis. [N Engl J Med.](http://www.ncbi.nlm.nih.gov/pubmed/?term=N+Engl+J+Med+371(17)%3A+1588-1598.) 2014 Oct 23; 371(17):1588-98. doi: 10.1056/NEJMoa1315817.
44. Mfinanga, S. G., B. J. Kirenga, et al. Early versus delayed initiation of highly active antiretroviral therapy for HIV-positive adults with newly diagnosed pulmonary tuberculosis (TB-HAART): a prospective, international, randomised, placebo-controlled trial. [Lancet Infect Dis.](http://www.ncbi.nlm.nih.gov/pubmed/?term=Lancet+Infect+Dis+14(7)%3A+563-571.) 2014 Jul; 14(7):563-71. doi: 10.1016/S1473-3099(14)70733-9.
45. Michelson, D., E. Snyder, et al. Safety and efficacy of suvorexant during 1-year treatment of insomnia with subsequent abrupt treatment discontinuation: a phase 3 randomised, double-blind, placebo-controlled trial. [Lancet Neurol.](http://www.ncbi.nlm.nih.gov/pubmed/?term=Lancet+Neurol+13(5)%3A+461-471.) 2014 May; 13(5):461-71. doi: 10.1016/S1474-4422(14)70053-5.
46. Mivumbi, V. N., S. E. Little, et al. Prophylactic ampicillin versus cefazolin for the prevention of post-cesarean infectious morbidity in Rwanda. [Int J Gynaecol Obstet.](http://www.ncbi.nlm.nih.gov/pubmed/?term=Int+J+Gynaecol+Obstet+124(3)%3A+244-247.) 2014 Mar; 124(3):244-7. doi: 10.1016/j.ijgo.2013.09.017.
47. Molyneux, E. M., K. Kawaza, et al. Glycerol and acetaminophen as adjuvant therapy did not affect the outcome of bacterial meningitis in Malawian children. [Pediatr Infect Dis J.](http://www.ncbi.nlm.nih.gov/pubmed/?term=Pediatr+Infect+Dis+J+33(2)%3A+214-216.) 2014 Feb; 33(2):214-6. doi: 10.1097/INF.0000000000000122.
48. Mosha, T. C. E., H. H. Laswai, et al. Efficacy of a low-dose ferric-EDTA in reducing iron deficiency anaemia among underfive children living in malaria-holoendemic district of mvomero, Tanzania. Tanzanian J Health Res 2014 April; 16(2):1-10. Doi: <http://dx.doi.org/10.4314/thrb.v16i2.2>
49. Muhumuza, S., A. Olsen, et al. Effectiveness of a pre-treatment snack on the uptake of mass treatment for schistosomiasis in Uganda: a cluster randomized trial. [PLoS Med.](http://www.ncbi.nlm.nih.gov/pubmed/?term=PLoS+Med+11(5)%3A+e1001640.) 2014 May 13; 11(5): e1001640. doi: 10.1371/journal.pmed.1001640.
50. Nankabirwa, J. I., B. Wandera, et al. Impact of intermittent preventive treatment with dihydroartemisinin- piperaquine on Malaria in Ugandan schoolchildren: A randomized, placebo-controlled trial. [Clin Infect Dis.](http://www.ncbi.nlm.nih.gov/pubmed/?term=Impact+of+intermittent+preventive+treatment+with+dihydroartemisinin-+piperaquine+on+Malaria+in+Ugandan+schoolchildren%3A+A+randomized%2C+placebo-controlled+trial) 2014 May; 58(10) :1404-12. doi: 10.1093/cid/ciu150.
51. Ntagirabiri, R., S. Harerimana, et al. Helicobacter pylori au Burundi : première évaluation de la prévalence en endoscopie et de l’éradication. J. Afr. Hépatol. Gastroentérol. 2014; 8:217-222. DOI 10.1007/s12157-014-0567-3.
52. Oguche, S., H. U. Okafor, et al. Efficacy of artemisinin-based combination treatments of uncomplicated falciparum malaria in under-five-year-old Nigerian children. [Am J Trop Med Hyg.](http://www.ncbi.nlm.nih.gov/pubmed/?term=Efficacy+of+artemisinin-based+combination+treatments+of+uncomplicated+falciparum+malaria+in+under-five-year-old+Nigerian+children) 2014 Nov; 91(5):925-35. doi: 10.4269/ajtmh.13-0248
53. Ogutu, B. R., K. O. Onyango, et al. Efficacy and safety of artemether-lumefantrine and dihydroartemisinin-piperaquine in the treatment of uncomplicated Plasmodium falciparum malaria in Kenyan children aged less than five years: results of an open-label, randomized, single-centre study. M[Malar J.](http://www.ncbi.nlm.nih.gov/pubmed/?term=Efficacy+and+safety+of+artemether-lumefantrine+and+dihydroartemisinin-piperaquine+in+the+treatment+of+uncomplicated+Plasmodium+falciparum+malaria+in+Kenyan+children+aged+less+than+five+years%3A+results+of+an+open-label%2C+randomized%2C+single-centre+study) 2014 Jan 28; 13:33. doi: 10.1186/1475-2875-13-33.
54. Olsen, M. F., A. Abdissa, et al. Effects of nutritional supplementation for HIV patients starting antiretroviral treatment: Randomised controlled trial in Ethiopia. [BMJ.](http://www.ncbi.nlm.nih.gov/pubmed/?term=Effects+of+nutritional+supplementation+for+HIV+patients+starting+antiretroviral+treatment%3A+Randomised+controlled+trial+in+Ethiopia) 2014 May 15; 348: g3187. doi: 10.1136/bmj. g3187.
55. Onyamboko, M. A., C. I. Fanello, et al. Randomized comparison of the efficacies and tolerabilities of three artemisinin-based combination treatments for children with acute Plasmodium falciparum malaria in the Democratic Republic of the Congo. [Antimicrob Agents Chemother.](http://www.ncbi.nlm.nih.gov/pubmed/?term=Randomized+comparison+of+the+efficacies+and+tolerabilities+of+three+artemisinin-based+combination+treatments+for+children+with+acute+Plasmodium+falciparum+malaria+in+the+Democratic+Republic+of+the+Congo) 2014 Sep; 58(9):5528-36. doi: 10.1128/AAC.02682-14.
56. Paton, N. I., C. Kityo, et al. Assessment of second-line antiretroviral regimens for HIV therapy in Africa. [N Engl J Med.](http://www.ncbi.nlm.nih.gov/pubmed/?term=N+Engl+J+Med+371(3)%3A+234-247.) 2014 Jul 17; 371(3):234-47. doi: 10.1056/NEJMoa1311274.
57. Petry, N., I. Egli, et al. Phytic acid concentration influences iron bioavailability from biofortified beans in Rwandese women with low iron status. [J Nutr.](http://www.ncbi.nlm.nih.gov/pubmed/?term=J+Nutr+144(11)%3A+1681-1687) 2014 Nov; 144(11):1681-7. doi: 10.3945/jn.114.192989.
58. Polis, C. B., G. F. Nakigozi, et al. Preference for Sayana Press versus intramuscular Depo-Provera among HIV-positive women in Rakai, Uganda: A randomized crossover trial. [Contraception.](http://www.ncbi.nlm.nih.gov/pubmed/?term=Preference+for+Sayana+Press+versus+intramuscular+Depo-Provera+among+HIV-positive+women+in+Rakai%2C+Uganda%3A+A+randomized+crossover+trial.) 2014 May; 89(5):385-95. doi: 10.1016/j.contraception.2013.11.008.
59. Price, T. J., M. Peeters, et al. Panitumumab versus cetuximab in patients with chemotherapy-refractory wild-type KRAS exon 2 metastatic colorectal cancer (ASPECCT): a randomised, multicentre, open-label, non-inferiority phase 3 study. [Lancet Oncol.](http://www.ncbi.nlm.nih.gov/pubmed/?term=Lancet+Oncol+15(6)%3A+569-579.) 2014 May; 15(6):569-79. doi: 10.1016/S1470-2045(14)70118-4.
60. Puls, R., J. Amin, et al. Efficacy of 400 mg efavirenz versus standard 600 mg dose in HIV-infected, antiretroviral-naive adults (ENCORE1): a randomised, double-blind, placebo-controlled, non-inferiority trial. [Lancet.](http://www.ncbi.nlm.nih.gov/pubmed/?term=Lancet+383(9927)%3A+1474-1482.) 2014 Apr 26; 383(9927):1474-82. doi: 10.1016/S0140-6736(13)62187-X.
61. Raal, F. J., N. Honarpour, et al. Inhibition of PCSK9 with evolocumab in homozygous familial hypercholesterolaemia (TESLA Part B): a randomised, double-blind, placebo-controlled trial. [Lancet.](http://www.ncbi.nlm.nih.gov/pubmed/?term=Lancet+385(9965)%3A+341-350.) 2015 Jan 24; 385(9965):341-50. doi: 10.1016/S0140-6736(14)61374-X.
62. Raal, F. J., E. A. Stein, et al. (2015). PCSK9 inhibition with evolocumab (AMG 145) in heterozygous familial hypercholesterolaemia (RUTHERFORD-2): a randomised, double-blind, placebo-controlled trial. [Lancet.](http://www.ncbi.nlm.nih.gov/pubmed/?term=Lancet+385(9965)%3A+331-340.) 2015 Jan 24; 385(9965):331-40. doi: 10.1016/S0140-6736(14)61399-4.
63. Rangaka, M. X., R. J. Wilkinson, et al. Isoniazid plus antiretroviral therapy to prevent tuberculosis: a randomised double-blind, placebo-controlled trial. [Lancet.](http://www.ncbi.nlm.nih.gov/pubmed/?term=Lancet+384(9944)%3A+682-690.) 2014 Aug 23; 384(9944):682-90. doi: 10.1016/S0140-6736(14)60162-8.
64. Reck, M., R. Kaiser, et al. Docetaxel plus nintedanib versus docetaxel plus placebo in patients with previously treated non-small-cell lung cancer (LUME-Lung 1): a phase 3, double-blind, randomised controlled trial. [Lancet Oncol.](http://www.ncbi.nlm.nih.gov/pubmed/?term=Lancet+Oncol+15(2)%3A+143-155.) 2014 Feb; 15(2):143-55. doi: 10.1016/S1470-2045(13)70586-2.
65. Ryan, K. N., K. B. Stephenson, et al. Zinc or albendazole attenuates the progression of environmental enteropathy: A randomized controlled trial. [Clin Gastroenterol Hepatol.](http://www.ncbi.nlm.nih.gov/pubmed/24462483) 2014 Sep; 12(9):1507-13. e1. doi: 10.1016/j.cgh.2014.01.024.
66. Shekalaghe, S., M. Rutaihwa, et al. Controlled human malaria infection of Tanzanians by intradermal injection of aseptic, purified, cryopreserved Plasmodium falciparum sporozoites. [Am J Trop Med Hyg.](http://www.ncbi.nlm.nih.gov/pubmed/?term=Am+J+Trop+Med+Hyg+91(3)%3A+471-480.) 2014 Sep; 91(3):471-80. doi: 10.4269/ajtmh.14-0119.
67. Sjouke, B., G. Langslet, et al. Eprotirome in patients with familial hypercholesterolaemia (the AKKA trial): A randomised, double-blind, placebo-controlled phase 3 study. [Lancet Diabetes Endocrinol.](http://www.ncbi.nlm.nih.gov/pubmed/?term=Eprotirome+in+patients+with+familial+hypercholesterolaemia+(the+AKKA+trial)%3A+A+randomised%2C+double-blind%2C+placebo-controlled+phase+3+study) 2014 Jun; 2(6):455-63. doi: 10.1016/S2213-8587(14)70006-3.
68. Speich, B., S. M. Ame, et al. Oxantel pamoate-albendazole for Trichuris trichiura infection. [N Engl J Med.](http://www.ncbi.nlm.nih.gov/pubmed/?term=N+Engl+J+Med+370(7)%3A+610-620.) 2014 Feb 13; 370(7):610-20. doi: 10.1056/NEJMoa1301956.
69. Thacher, T. D., P. R. Fischer, et al. Vitamin D treatment in calcium-deficiency rickets: a randomised controlled trial. [Arch Dis Child.](http://www.ncbi.nlm.nih.gov/pubmed/?term=Arch+Dis+Child+99(9)%3A+807-811.) 2014 Sep; 99(9):807-11. doi: 10.1136/archdischild-2013-305275.
70. Sundy, J. S., H. R. Schumacher, et al. Rilonacept for gout flare prevention in patients receiving uric acid-lowering therapy: Results of RESURGE, a phase III, international safety study. [J Rheumatol.](http://www.ncbi.nlm.nih.gov/pubmed/?term=Rilonacept+for+gout+flare+prevention+in+patients+receiving+uric+acid-lowering+therapy%3A+Results+of+RESURGE%2C+a+phase+III%2C+international+safety+study.) 2014 Aug; 41(8):1703-11. doi: 10.3899/jrheum.131226.
71. Thakwalakwa, C., A. Phiri, et al. Growth and HIV-free survival of HIV-exposed infants in Malawi: a randomized trial of two complementary feeding interventions in the context of maternal antiretroviral therapy. [J Acquir Immune Defic Syndr.](http://www.ncbi.nlm.nih.gov/pubmed/?term=J+Acquir+Immune+Defic+Syndr+66(2)%3A+181-187) 2014 Jun 1; 66(2):181-7. doi: 10.1097/QAI.0000000000000150.
72. Tinto, H., S. Diallo, et al. Effectiveness of artesunate-amodiaquine vs. artemether-lumefantrine for the treatment of uncomplicated falciparum malaria in Nanoro, Burkina Faso: a non-inferiority randomised trial. [Trop Med Int Health.](http://www.ncbi.nlm.nih.gov/pubmed/?term=Trop+Med+Int+Health+19(4)%3A+469-475.) 2014 Apr; 19(4):469-75. doi: 10.1111/tmi.12274.
73. Westen, E. H., P. R. Kolk, et al. Single-dose compared with multiple day antibiotic prophylaxis for cesarean section in low-resource settings, a randomized controlled, noninferiority trial. [Acta Obstet Gynecol Scand.](http://www.ncbi.nlm.nih.gov/pubmed/?term=Single-dose+compared+with+multiple+day+antibiotic+prophylaxis+for+cesarean+section+in+low-resource+settings%2C+a+randomized+controlled%2C+noninferiority+trial) 2015 Jan; 94(1):43-9. doi: 10.1111/aogs.12517.
74. Yeka, A., V. Lameyre, et al. Efficacy and safety of fixed-dose artesunate-amodiaquine vs. artemether-lumefantrine for repeated treatment of uncomplicated malaria in Ugandan children. [PLoS One.](http://www.ncbi.nlm.nih.gov/pubmed/?term=Efficacy+and+safety+of+fixed-dose+artesunate-amodiaquine+vs.+artemether-lumefantrine+for+repeated+treatment+of+uncomplicated+malaria+in+Ugandan+children.) 2014 Dec 1; 9(12): e113311. doi: 10.1371/journal.pone.0113311.
75. **Non-pharmacological treatments**

B.1 Participative interventions

1. Adam, M. B. Effectiveness trial of community-based I Choose Life-Africa human immunodeficiency virus prevention program in Kenya. [Am J Trop Med Hyg.](http://www.ncbi.nlm.nih.gov/pubmed/?term=Effectiveness+trial+of+community-based+I+Choose+Life-Africa+human+immunodeficiency+virus+prevention+program+in+Kenya) 2014 Sep; 91(3):645-8. doi: 10.4269/ajtmh.14-0141.
2. Althabe, F., J. M. Belizan, et al. A population-based, multifaceted strategy to implement antenatal corticosteroid treatment versus standard care for the reduction of neonatal mortality due to preterm birth in low-income and middle-income countries: the ACT cluster-randomised trial. [Lancet.](http://www.ncbi.nlm.nih.gov/pubmed/?term=A+population-based%2C+multifaceted+strategy+to+implement+antenatal+corticosteroid+treatment+versus+standard+care+for+the+reduction+of+neonatal+mortality+due+to+preterm+birth+in+low-income+and+middle-income+countries%3A+the+ACT+cluster-randomised+trial) 2015 Feb 14; 385(9968):629-39. doi: 10.1016/S0140-6736(14)61651-2.
3. Anyachukwu, C. C. and O. K. K. Onyeso. Efficacy of adjunct (laser) therapy to topical agents among Southern Nigerian acne vulgaris patients. Acupuncture and Related Therapies 2 (2014) 66–70.
4. Baatjies, R., T. Meijster, et al. Effectiveness of interventions to reduce flour dust exposures in supermarket bakeries in South Africa. [Occup Environ Med.](http://www.ncbi.nlm.nih.gov/pubmed/?term=Effectiveness+of+interventions+to+reduce+flour+dust+exposures+in+supermarket+bakeries+in+South+Africa) 2014 Dec; 71(12):811-8. doi: 10.1136/oemed-2013-101971.
5. Betancourt, T. S., R. McBain, et al. A behavioral intervention for war-affected youth in Sierra Leone: A randomized controlled trial. [J Am Acad Child Adolesc Psychiatry.](http://www.ncbi.nlm.nih.gov/pubmed/?term=A+behavioral+intervention+for+war-affected+youth+in+Sierra+Leone%3A+A+randomized+controlled+trial.) 2014 Dec; 53(12):1288-97. doi: 10.1016/j.jaac.2014.09.011.
6. Bigna, J. J., J. J. Noubiap, et al. Effect of mobile phone reminders on follow-up medical care of children exposed to or infected with HIV in Cameroon (MORE CARE): a multicentre, single-blind, factorial, randomised controlled trial. [Lancet Infect Dis.](http://www.ncbi.nlm.nih.gov/pubmed/?term=Lancet+Infect+Dis+14(7)%3A+600-608.) 2014 Jul; 14(7):600-8. doi: 10.1016/S1473-3099(14)70741-8.
7. Botha, U. A., L. Koen, et al. The rise of assertive community interventions in South Africa: A randomized control trial assessing the impact of a modified assertive intervention on readmission rates; a three-year follow-up. [BMC Psychiatry.](http://www.ncbi.nlm.nih.gov/pubmed/?term=The+rise+of+assertive+community+interventions+in+South+Africa%3A+A+randomized+control+trial+assessing+the+impact+of+a+modified+assertive+intervention+on+readmission+rates%3B+a+three+year+follow-up) 2014 Feb 27; 14:56. doi: 10.1186/1471-244X-14-56.
8. Bruxvoort, K., C. Festo, et al. Cluster randomized trial of text message reminders to retail staff in tanzanian drug shops dispensing artemether-lumefantrine: Effect on dispenser knowledge and patient adherence. [Am J Trop Med Hyg.](http://www.ncbi.nlm.nih.gov/pubmed/?term=Cluster+randomized+trial+of+text+message+reminders+to+retail+staff+in+tanzanian+drug+shops+dispensing+artemether-lumefantrine%3A+Effect+on+dispenser+knowledge+and+patient+adherence) 2014 Oct; 91(4):844-53. doi: 10.4269/ajtmh.14-0126.
9. Constant, D., K. Tolly, et al. Mobile phone messages to provide support to women during the home phase of medical abortion in South Africa: A randomised controlled trial. [Contraception.](http://www.ncbi.nlm.nih.gov/pubmed/?term=Mobile+phone+messages+to+provide+support+to+women+during+the+home+phase+of+medical+abortion+in+South+Africa%3A+A+randomised+controlled+trial) 2014 Sep; 90(3):226-33. doi: 10.1016/j.contraception.2014.04.009.
10. Deressa, W., Y. Y. Yihdego, et al. Effect of combining mosquito repellent and insecticide treated net on malaria prevalence in Southern Ethiopia: a cluster-randomised trial. [Parasit Vectors.](http://www.ncbi.nlm.nih.gov/pubmed/?term=Effect+of+combining+mosquito+repellent+and+insecticide+treated+net+on+malaria+prevalence+in+Southern+Ethiopia%3A+a+cluster-randomised+trial) 2014 Mar 28; 7:132. doi: 10.1186/1756-3305-7-132.
11. Desrochers, R. E., K. Siekmans, et al. Effectiveness of post-campaign, door-to-door, hang-up, and communication interventions to increase long-lasting, insecticidal bed net utilization in Togo (2011-2012): A cluster randomized, control trial. [Malar J.](http://www.ncbi.nlm.nih.gov/pubmed/?term=Effectiveness+of+post-campaign%2C+door-to-door%2C+hang-up%2C+and+communication+interventions+to+increase+long-lasting%2C+insecticidal+bed+net+utilization+in+Togol) 2014 Jul 9; 13:260. doi: 10.1186/1475-2875-13-260.
12. Ezema, C. I., A. A. Onwunali, et al. Effect of aerobic exercise training on cardiovascular parameters and CD4 cell count of people living with human immunodeficiency virus/acquired immune deficiency syndrome: a randomized controlled trial. [Niger J Clin Pract.](http://www.ncbi.nlm.nih.gov/pubmed/?term=Niger+J+Clin+Pract+17(5)%3A+543-548.) 2014 Sep-Oct; 17(5):543-8. doi: 10.4103/1119-3077.141414.
13. Flax, V. L., M. Negerie, et al. Integrating group counseling, cell phone messaging, and participant-generated songs and dramas into a microcredit program increases Nigerian women's adherence to international breastfeeding recommendations. [J Nutr.](http://www.ncbi.nlm.nih.gov/pubmed/?term=J+Nutr+144(7)%3A+1120-1124.) 2014 Jul; 144(7):1120-4. doi: 10.3945/jn.113.190124.
14. Groome, M. J., S. S. Moon, et al. Effect of breastfeeding on immunogenicity of oral live-attenuated human rotavirus vaccine: A randomized trial in HIV-uninfected infants in Soweto, South Africa. [Bull World Health Organ.](http://www.ncbi.nlm.nih.gov/pubmed/?term=Effect+of+breastfeeding+on+immunogenicity+of+oral+live-attenuated+human+rotavirus+vaccine%3A+A+randomized+trial+in+HIV-uninfected+infants+in+Soweto%2C+South+Africa) 2014 Apr 1; 92(4):238-45. doi: 10.2471/BLT.13.128066.
15. Ibinda, F., C. K. Mbuba, et al. Evaluation of Kilifi epilepsy education programme: a randomized controlled trial. [Epilepsia.](http://www.ncbi.nlm.nih.gov/pubmed/?term=Epilepsia+55(2)%3A+344-352.) 2014 Feb; 55(2):344-52. doi: 10.1111/epi.12498.
16. Jacob, N., F. Neuner, et al. Dissemination of psychotherapy for trauma spectrum disorders in postconflict settings: A randomized controlled trial in Rwanda. [Psychother Psychosom.](http://www.ncbi.nlm.nih.gov/pubmed/?term=Dissemination+of+psychotherapy+for+trauma+spectrum+disorders+in+postconflict+settings%3A+A+randomized+controlled+trial+in+Rwanda) 2014; 83(6):354-63. doi: 10.1159/000365114.
17. Jemmott, I. J., L. S. Jemmott, et al. Theory-based behavioral intervention increases self-reported physical activity in South African men: A cluster-randomized controlled trial. [Prev Med.](http://www.ncbi.nlm.nih.gov/pubmed/24736094) 2014 Jul; 64:114-20. doi: 10.1016/j.ypmed.2014.04.012.
18. Jones, D., D. Kashy, et al. Risk reduction among HIV-seroconcordant and -discordant couples: The Zambia NOW2 intervention. [AIDS Patient Care STDS.](http://www.ncbi.nlm.nih.gov/pubmed/?term=AIDS+Patient+Care+STDS+28(8)%3A+433-441.) 2014 Aug; 28(8):433-41. doi: 10.1089/apc.2014.0039.
19. Keogh, S. C., K. Fry, et al. Vocal local versus pharmacological treatments for pain management in tubal ligation procedures in rural Kenya: a non-inferiority trial. [BMC Womens Health.](http://www.ncbi.nlm.nih.gov/pubmed/?term=Vocal+local+versus+pharmacological+treatments+for+pain+management+in+tubal+ligation+procedures+in+rural+Kenya%3A+a+non-inferiority+trial) 2014 Feb 4; 14:21. doi: 10.1186/1472-6874-14-21.
20. L'Engle, K. L., P. Mwarogo, et al. A randomized controlled trial of a brief intervention to reduce alcohol use among female sex workers in Mombasa, Kenya. [J Acquir Immune Defic Syndr.](http://www.ncbi.nlm.nih.gov/pubmed/?term=J+Acquir+Immune+Defic+Syndr+67(4)%3A+446-453.) 2014 Dec 1; 67(4):446-53. doi: 10.1097/QAI.0000000000000335.
21. Lund, S., B. B. Nielsen, et al. Mobile phones improve antenatal care attendance in Zanzibar: a cluster randomized controlled trial. [BMC Pregnancy Childbirth.](http://www.ncbi.nlm.nih.gov/pubmed/24438517) 2014 Jan 17; 14:29. doi: 10.1186/1471-2393-14-29.
22. MacPherson, P., D. G. Lalloo, et al. Effect of optional home initiation of HIV care following HIV self-testing on antiretroviral therapy initiation among adults in Malawi: a randomized clinical trial. [JAMA.](http://www.ncbi.nlm.nih.gov/pubmed/?term=JAMA+312(4)%3A+372-379.) 2014 Jul 23-30; 312(4):372-9. doi: 10.1001/jama.2014.6493.
23. Maman, S., D. Moodley, et al. Efficacy of enhanced HIV counseling for risk reduction during pregnancy and in the postpartum period: a randomized controlled trial. [PLoS One.](http://www.ncbi.nlm.nih.gov/pubmed/?term=PLoS+One+9(5)%3A+e97092.) 2014 May 13; 9(5): e97092. doi: 10.1371/journal.pone.0097092.
24. Mbacham, W. F., L. Mangham-Jefferies, et al. Basic or enhanced clinician training to improve adherence to malaria treatment guidelines: A cluster-randomised trial in two areas of Cameroon. [Lancet Glob Health.](http://www.ncbi.nlm.nih.gov/pubmed/?term=A+cluster-randomised+trial+in+two+areas+of+Cameroon) 2014 Jun; 2(6): e346-58. doi: 10.1016/S2214-109X(14)70201-3.
25. Mbada, C. E., O. Ayanniyi, et al. Influence of Mckenzie protocol and two modes of endurance exercises on health-related quality of life of patients with long-term mechanical low-back pain. [Pan Afr Med J.](http://www.ncbi.nlm.nih.gov/pubmed/?term=Influence+of+Mckenzie+protocol+and+two+modes+of+endurance+exercises+on+health-related+quality+of+life+of+patients+with+long-term+mechanical+low-back+pain) 2014 Jan 18; 17 Suppl 1:5. doi: 10.11694/pamj.supp.2014.17.1.2950.
26. Mertens, J. R., C. L. Ward, et al. Effectiveness of nurse-practitioner-delivered brief motivational intervention for young adult alcohol and drug use in primary care in South Africa: A randomized clinical trial. [Alcohol.](http://www.ncbi.nlm.nih.gov/pubmed/?term=Effectiveness+of+nurse-practitioner-delivered+brief+motivational+intervention+for+young+adult+alcohol+and+drug+use+in+primary+care+in+South+Africa%3A+A+randomized+clinical+trial) 2014 Jul-Aug; 49(4):430-8. doi: 10.1093/alcalc/agu030.
27. Nkengfack, G. N., J. N. Torimiro, et al. Effects of an HIV-Care-Program on immunological parameters in HIV-positive patients in Yaounde, Cameroon: a cluster-randomized trial. [Int J Public Health.](http://www.ncbi.nlm.nih.gov/pubmed/?term=Int+J+Public+Health+59(3)%3A+509-517.) 2014 Jun; 59(3):509-17. doi: 10.1007/s00038-014-0547-9.
28. Olaleye, O. A., T. K. Hamzat, et al. (2014). Stroke rehabilitation: should physiotherapy intervention be provided at a primary health care centre or the patients' place of domicile? [Disabil Rehabil.](http://www.ncbi.nlm.nih.gov/pubmed/?term=Disabil+Rehabil+36(1)%3A+49-54.) 2014; 36(1):49-54. doi: 10.3109/09638288.2013.777804.
29. Osoti, A. O., G. John-Stewart, et al. Home visits during pregnancy enhance male partner HIV counselling and testing in Kenya: a randomized clinical trial. [AIDS.](http://www.ncbi.nlm.nih.gov/pubmed/?term=Home+visits+during+pregnancy+enhance+male+partner+HIV+counselling+and+testing+in+Kenya%3A+a+randomized+clinical+trial) 2014 Jan 2; 28(1):95-103. doi: 10.1097/QAD.0000000000000023.
30. Penfold, S., F. Manzi, et al. Effect of home-based counselling on newborn care practices in southern tanzania one year after implementation: A cluster-randomised controlled trial. [BMC Pediatr.](http://www.ncbi.nlm.nih.gov/pubmed/?term=Effect+of+home-based+counselling+on+newborn+care+practices+in+southern+tanzania+one+year+after+implementation%3A+A+cluster-randomised+controlled+trial) 2014 Jul 22; 14:187. doi: 10.1186/1471-2431-14-187.
31. Raifman, J. R. G., H. E. Lanthorn, et al. The impact of text message reminders on adherence to antimalarial treatment in northern ghana: A randomized trial. [PLoS One.](http://www.ncbi.nlm.nih.gov/pubmed/?term=The+impact+of+text+message+reminders+on+adherence+to+antimalarial+treatment+in+northern+ghana%3A+A+randomized+trial) 2014 Oct 28; 9(10): e109032. doi: 10.1371/journal.pone.0109032.
32. Richter, L., M. J. Rotheram-Borus, et al. Pregnant women living with HIV (WLH) supported at clinics by peer WLH: a cluster randomized controlled trial. [AIDS Behav.](http://www.ncbi.nlm.nih.gov/pubmed/?term=AIDS+Behav+18(4)%3A+706-715.) 2014 Apr; 18(4):706-15. doi: 10.1007/s10461-014-0694-2.
33. Rosa, G., F. Majorin, et al. Assessing the impact of water filters and improved cook stoves on drinking water quality and household air pollution: A randomised controlled trial in Rwanda. [PLoS One.](http://www.ncbi.nlm.nih.gov/pubmed/?term=Assessing+the+impact+of+water+filters+and+improved+cook+stoves+on+drinking+water+quality+and+household+air+pollution%3A+A+randomised+controlled+trial+in+Rwanda) 2014 Mar 10; 9(3): e91011. doi: 10.1371/journal.pone.0091011.
34. Sossauer, G., M. Zbinden, et al. Impact of an educational intervention on women's knowledge and acceptability of human papillomavirus self-sampling: A randomized controlled trial in cameroon. [PLoS One.](http://www.ncbi.nlm.nih.gov/pubmed/25333793) 2014 Oct 15; 9(10): e109788. doi: 10.1371/journal.pone.0109788.
35. Sumner, S. A., A. J. Pallangyo, et al. Effect of free distribution of safety equipment on usage among motorcycle-taxi drivers in Tanzania - A cluster randomised controlled trial. [Injury.](http://www.ncbi.nlm.nih.gov/pubmed/?term=Effect+of+free+distribution+of+safety+equipment+on+usage+among+motorcycle-taxi+drivers+in+Tanzania+-+A+cluster+randomised+controlled+trial) 2014 Nov; 45(11):1681-6. doi: 10.1016/j.injury.2014.04.034.
36. Tiono, A. B., D. T. Kangoye, et al. Malaria incidence in children in South-West Burkina Faso: Comparison of active and passive case detection methods. [PLoS One.](http://www.ncbi.nlm.nih.gov/pubmed/?term=Malaria+incidence+in+children+in+South-West+Burkina+Faso%3A+Comparison+of+active+and+passive+case+detection+methods) 2014 Jan 24; 9(1): e86936. doi: 10.1371/journal.pone.0086936.
37. Tol, W. A., I. H. Komproe, et al. School-based mental health intervention for children in war-affected Burundi: A cluster randomized trial. [BMC Med.](http://www.ncbi.nlm.nih.gov/pubmed/?term=.+School-based+mental+health+intervention+for+children+in+war-affected+Burundi%3A+A+cluster+randomized+trial) 2014 Apr 1; 12:56. doi: 10.1186/1741-7015-12-56.
38. Tomlinson, M., T. Doherty, et al. Goodstart: a cluster randomised effectiveness trial of an integrated, community-based package for maternal and newborn care, with prevention of mother-to-child transmission of HIV in a South African township. [Trop Med Int Health.](http://www.ncbi.nlm.nih.gov/pubmed/?term=Trop+Med+Int+Health+19(3)%3A+256-266.) 2014 Mar; 19(3):256-66. doi: 10.1111/tmi.12257.
39. West, P. A., N. Protopopoff, et al. Indoor residual spraying in combination with insecticide-treated nets compared to insecticide-treated nets alone for protection against malaria: a cluster randomised trial in Tanzania. [PLoS Med.](http://www.ncbi.nlm.nih.gov/pubmed/?term=PLoS+Med+11(4)%3A+e1001630.) 2014 Apr 15; 11(4): e1001630. doi: 10.1371/journal.pmed.1001630.
40. Yan, H., A. Prista, et al. Effect of aerobic training on glucose control and blood pressure in T2DDM East African males. [ISRN Endocrinol.](http://www.ncbi.nlm.nih.gov/pubmed/?term=Effect+of+aerobic+training+on+glucose+control+and+blood+pressure+in+T2DDM+East+African+males) 2014 Mar 4; 2014:864897. doi: 10.1155/2014/864897.

B.2 Procedures (surgery, therapeutic strategies and devices)

- 1. Nicol, A. J., P. H. Navsaria, et al. Sternotomy or drainage for a hemopericardium after penetrating trauma: a randomized controlled trial. [Ann Surg.](http://www.ncbi.nlm.nih.gov/pubmed/?term=Ann+Surg+259(3)%3A+438-442.) 2014 Mar; 259(3):438-42. doi: 10.1097/SLA.0b013e31829069a1.
  2. Chigbu, C. O. and A. K. Onyebuchi. See-and-treat management of high-grade squamous intraepithelial lesions in a resource-constrained African setting. [Int J Gynaecol Obstet.](http://www.ncbi.nlm.nih.gov/pubmed/?term=Int+J+Gynaecol+Obstet+124(3)%3A+204-206.) 2014 Mar; 124(3):204-6. doi: 10.1016/j.ijgo.2013.07.040.
  3. Harilall, Y., J. K. Adam, et al. The effect of optimising cerebral tissue oxygen saturation on markers of neurological injury during coronary artery bypass graft surgery. [Heart Lung Circ.](http://www.ncbi.nlm.nih.gov/pubmed/?term=Heart+Lung+Circ+23(1)%3A+68-74.) 2014 Jan; 23(1):68-74. doi: 10.1016/j.hlc.2013.07.002.
  4. Ugwu, E. O., S. N. Obi, et al. Membrane stripping to prevent post-term pregnancy in Enugu, Nigeria: a randomized controlled trial. [Arch Gynecol Obstet.](http://www.ncbi.nlm.nih.gov/pubmed/?term=Arch+Gynecol+Obstet+289(1)%3A+29-34.) 2014 Jan; 289(1):29-34. doi: 10.1007/s00404-013-2918-5.
  5. Millard, P. S., H. R. Wilson, et al. Rapid, minimally invasive adult voluntary male circumcision: a randomised trial of Unicirc, a novel disposable device. [S Afr Med J.](http://www.ncbi.nlm.nih.gov/pubmed/?term=S+Afr+Med+J+104(1)%3A+52-57.) 2013 Nov 20; 104(1):52-7. doi: 10.7196/samj.7357.
  6. Reznik, Y., O. Cohen, et al. Insulin pump treatment compared with multiple daily injections for treatment of type 2 diabetes (OpT2mise): a randomised open-label controlled trial. [Lancet.](http://www.ncbi.nlm.nih.gov/pubmed/?term=Lancet+384(9950)%3A+1265-1272.) 2014 Oct 4; 384(9950):1265-72. doi: 10.1016/S0140-6736(14)61037-0.
  7. Sokal, D. C., P. S. Li, et al. Randomized controlled trial of the shang ring versus conventional surgical techniques for adult male circumcision: safety and acceptability. [J Acquir Immune Defic Syndr.](http://www.ncbi.nlm.nih.gov/pubmed/?term=Randomized+controlled+trial+of+the+shang+ring+versus+conventional+surgical+techniques+for+adult+male+circumcision%3A+safety+and+acceptability) 2014 Apr 1; 65(4):447-55. doi: 10.1097/QAI.0000000000000061.
